# Strangers in a strange land: mapping household and neighbourhood associations with improved wellbeing outcomes in Accra, Ghana

**DOI:** 10.1016/j.cities.2023.104584

**Published:** 2023-09-28

**Authors:** Alicia C. Cavanaugha, Jill C. Baumgartner, Honor Bixby, Alexandra M. Schmidt, Samuel Agyei-Mensah, Samuel K. Annim, Jacqueline Anum, Raphael Arku, James Bennett, Frans Berkhout, Majid Ezzati, Samilia E. Mintah, George Owusu, Jacob Doku Tetteh, Brian E. Robinson

**Affiliations:** a Department of Geography, McGill University, Montreal, QC, Canada; b Department of Epidemiology, Biostatistics and Occupational Health, McGill University, Montreal, QC, Canada; c Department of Geography, University of Ghana, Legon, Ghana; d Ghana Statistical Service, Accra, Ghana; e Department of Environmental Health Sciences, University of Massachusetts, USA; f Department of Epidemiology and Biostatistics, Imperial College London, London, UK; g Department of Geography, King’s College London, London, UK

**Keywords:** inequality, poverty, Africa, neighbourhood effects, segregation, well-being

## Abstract

Urban poverty is not limited to informal settlements, rather it extends throughout cities, with the poor and affluent often living in close proximity. Using a novel dataset derived from the full Ghanaian Census, we investigate how neighbourhood versus household socio-economic status (SES) relates to a set of household development outcomes (related to housing quality, energy, water and sanitation, and information technology) in Accra, Ghana. We then assess “stranger” households’ outcomes within neighbourhoods: do poor households fare better in affluent neighbourhoods, and are affluent households negatively impacted by being in poor neighbourhoods? Through a simple generalized linear model we estimate the variance components associated with household and neighbourhood status for our outcome measures. Household SES is more closely associated with 13 of the 16 outcomes assessed compared to the neighbourhood average SES. Second, for 9 outcomes poor households in affluent areas fair better, and the affluent in poor areas are worse off. For two outcomes, poor households have worse outcomes in affluent areas, and the affluent have better outcomes in poor areas, on average. For three outcomes “stranger” households do worse in strange neighbourhoods. We discuss implications for mixed development and how to direct resources through households versus location-based targets.

## Introduction

The global development agenda has focused on poverty reduction since its inception. Adequate financial resources allow households to protect themselves from risks and make investment choices that capitalize on endowments, skills, or other natural advantages and make choices, sometimes implying trade-offs, to maximize utility given their budget constraints. These may include investments in education, asset accumulation, or other forms of capital that help buffer risk and improve overall well-being, but households with limited means may also be forced to make trade-offs in meeting basic needs and making such investments. A dominant focus of development policy and the current Sustainable Development Goals (i.e., SDG 10) has centred around how to support such households and break cycles of poverty and reduce overall inequalities, particularly in Lower Middle-Income Countries (LMIC).

In addition to this focus on household-level poverty, a broad literature across a myriad of disciplines now also investigates how one’s location and the neighbourhood environment can have dramatic and independent effects on household living conditions, well-being, and health outcomes (Sampson, 2008, 2011; Reardon & Bischoff, 2011; Parks et al., 2014; Ansong et al., 2015; Fowler & Kleit, 2015; Montgomery & Hewitt, 2015; Rothwell & Massey, 2015; Doiron et al., 2020; Mah et al., 2022). Recent reviews of ‘neighbourhood effects’ suggest a household’s location can impact outcomes through both physical and social contextual factors beyond just income. The physical environment includes commons that everyone shares (e.g. air quality, soils, etc.), general living and working conditions (e.g. quality of housing stock, reliable and safe employment), or access to services (e.g. education, transport, health, sanitation) (Fowler & Kleit, 2015; Wen et al., 2003). Social environments can indicate the cohesiveness of community networks, levels of crime, and how a neighbourhood is perceived by the wider community (Macintyre et al., 2002; Rothwell & Massey, 2015). These neighbourhood characteristics often accumulate through multiple individual choices that can sort individuals and households into particular neighbourhoods (Brock & Durlauf, 2001; Salhab et al., 2018; Walks, 2014; Wessel, 2022).

Yet the literature on poverty and neighbourhood effects remains relatively siloed. For example, neighbourhood context has been shown to mediate access to jobs, earning potential, education, or limit access to public and health services, but the influence of household SES relative to neighbourhood conditions are not directly compared (Massey & Denton, 1993; Smets & Salman, 2008; Ludwig et al., 2012; Rothwell & Massey, 2015; Chetty et al., 2016). Neighbourhoods with larger middle- or upper-class populations are associated with greater access to material and social resources that support local institutions (Wilson, 1987; Browning & Cagney, 2003), implying poorer households located in affluent communities may be better off than a poor household in a poor community, and affluent households may be worse off when they are in a poor neighbourhood, though these are not directly assessed. Massey (2001, 46) succinctly suggested that “living in a neighbourhood of concentrated poverty accentuates and exacerbates whatever disadvantages come from living in a poor family, and that living in a neighbourhood of concentrated affluence reinforces and strengthens the advantages of coming from an economically privileged family.”

Moreover, the literature on spatial inequalities and segregation sheds little light on how and when the neighbourhood context matters relative to household factors like income (Sharkey & Faber, 2016). Segregation alone has received a great deal of attention in higher- and lower-income country (HIC and LMIC, respectively) cities (van Ham et al., 2021), but the literature on segregation is generally silent on interactions with household factors. Although individual, household, and neighbourhood effects have been well-examined in hierarchical frameworks, these typically do not focus on interaction (Diez Roux, 2001; Sharkey & Faber, 2016; Chetty et al., 2022). Of the few studies we find that do, they focus on HICs and examine health outcomes (e.g., Rachele et al., 2019; Kim & Cubbin, 2020). Another study in Ghana used a variance components model to explore the interaction between neighbourhood context and household education in determining aspects of health knowledge (Andrzejewski, et al., 2009). Still, understanding the magnitude and extent to which household versus neighbourhood pathways interact and affect important development outcomes could have important implications for designing place-based or household targeted policies (Partridge & Rickman, 2008; Ludwig et al., 2012).

In this paper we ask how much does neighbourhood versus household socio-economic status (SES) affect household outcomes? We disentangle these effects through use of a dataset derived from the full 100% Ghanaian census. In collaboration with the Ghana Statistical Service, our unique dataset allows us to estimate the full distribution of SES and at the Enumeration Area (EA) (on average about 10,000m^2^, similar to a US census block) for Accra, Ghana. We investigate outcomes related to living standards and access to information among “stranger” households within neighbourhoods: do poor households fare better in affluent neighbourhoods, and are affluent households negatively impacted by being in poor neighbourhoods? To descriptively assess these differences, we use a simple variance-components regression framework to separate the independent associations between households versus neighbourhood SES on household-level living standard outcomes. We find a number of household conditions are primarily associated with household SES, while fewer are associated more closely with location SES. However, a key contribution from this work is that even for outcomes where SES matters more, location interactions with household SES can still have major implications for living conditions. This provides evidence about the heterogeneity of neighbourhoods in Accra, and the implications of the poor living in better neighbourhoods. Given the government’s push for mixed development to encourage the creation of economically diverse communities, we seek to understand whether this type of planning benefits all households or whether it will need to be paired with more targeted interventions (Joseph et al., 2022).

### Case Study: Accra, Ghana

Accra, the political and economic capital of Ghana, is one of Africa’s fastest growing cities. Recent economic growth in the Accra Metropolitan Area (hereafter simply referred to as Accra) is driven by growth in the service sector – both in high-income jobs in the finance, insurance, and real estate (FIRE) and information and communication technology (ICT) sectors, and low-income informal employment. Despite growth in high-skill formal occupations, Ghana’s economy remains quite informal – in Greater Accra, informal labour accounts for 73% of all workers employed in services (Aryeetey & Baah-Boateng, 2016). Growth in these sectors contributes to a widening wage structure, increasing metropolitan inequality across the city (Borel-Saladin & Crankshaw, 2009; Aryeetey & Baah-Boateng, 2016).

Some areas developed under colonial urban planning projects that were originally intended for civil servants and lease to European businesses later became home for Ghanaian civil servants post-independence (Agyei-Mensah & Owusu, 2010). Other areas were more recently developed, with many high-end developments built upon efforts to stimulate domestic growth (Gaisie et al., 2019), though many neighbourhoods remain highly mixed (Asiedu & Arku 2009; Gaisie et al., 2019).

In addition to planning and policy decisions operating at a macro scale, the socio-spatial organization of Accra is also shaped by individuals making choices to select into areas based on access to employment, and social connections. A primary consideration for households is the rising costs of land, construction, and financing. For some, this pushes them from the formal housing market into overcrowded, poorly serviced informal settlements (Boamah, 2010; Gaisie et al., 2019). In 2009, around 58% of the population of Accra resided in overcrowded informal settlements (UN-Habitat, 2009). In these areas housing is often lived in rent-free (usually unowned housing or living with family members), though formal ownership or renting of housing is not uncommon. In poor and informal settlements, households often do not have access to common public services like water and sanitation infrastructure, and generally cannot afford private substitutes (Boamah, 2010; Obeng-Odoom, 2011). Still, informal settlements and poor living conditions are not synonymous. Neighborhood characteristics in part culminate from individual choices around housing materials, lighting, fuel choices, and information technology, though these can also be constrained by cost, availability, access, and supply (Danso-Wiredu, 2018; MacTavish et al., 2023). Additionally, informal jobs often emerge in public spaces and along major transit corridors (Oosterbaan et al., 2012), sometimes bringing informal housing settlements along with them.

Thus while areas of concentrated affluence and poverty surely exist in Accra, poor, middle-class, and affluent households often co-locate. Even in exclusive residential areas (i.e., gated communities), less well-off residents can live nearby (Asiedu & Arku, 2009). Despite the government’s encouragement of mixed income development, it is unclear whether living in an affluent neighbourhood will benefit households that are less well off or lead to worse outcomes for outgroup households. By the same token, in poor neighbourhoods, it is uncertain how atypical affluence might buffer a better-off household from local conditions of poverty and deprivation.

**Figure 1:**
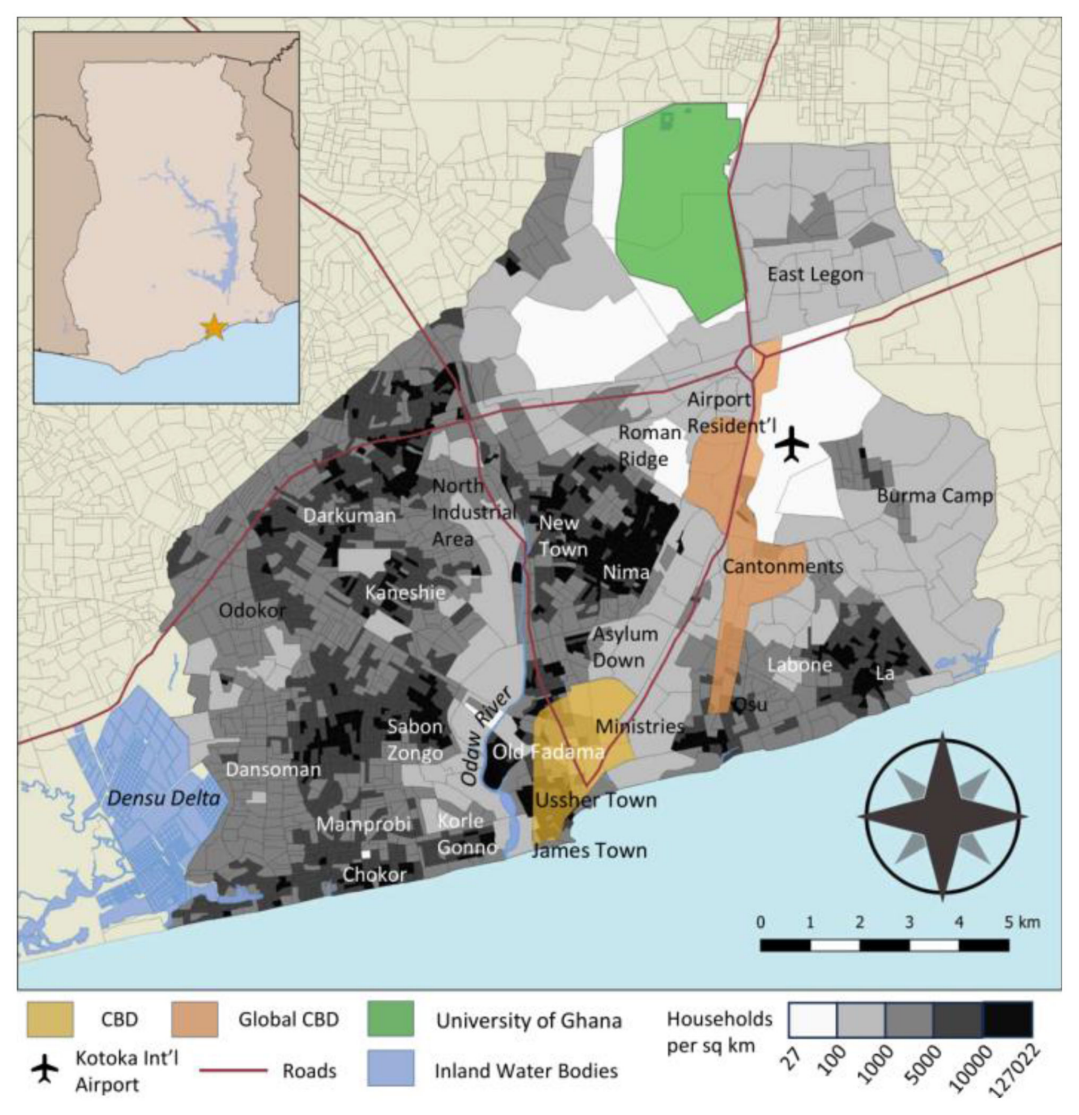
Housing Density and Accra Key Locations. Adapted from Gaisie et al. (2019) and Accra Planning and Development Programme (1990).

Spatially, the coastal center of Accra (Figure 1) is a European-style central business district (CBD) (Accra Planning and Development Programme 1990) that originally served the port. To the north by the Odaw River a market district emerged (Grant & Yankson, 2003), and a more globally-focused CBD emerged along major roads near the newly-developed Airport City and Accra Mall south to Osu (Grant & Nijman, 2002; Gaisie et al., 2019). The government has encouraged mixed-use development in this district, in part due to the rigidities of the old CBD (Oosterbaan et al., 2012). The density of households and populations varies dramatically across the city as well, with population clustered between the Densu Delta and surrounding the Odaw River floodplain, with pockets of communities (e.g., Osu, La, Burma Camp, Legon) scattered in the lower densityeast. Of the population clusters shown (Figure 1), Old Fadama (Agbogbloshie), Nima, James Town, Chorkor, Sabon Zongo, and La are major informal settlements.

Accra is vulnerable to many of the issues that attend unplanned development such as increased urban poverty, rising distributional and spatial inequality, and environmental degradation (Awumbila et al., 2014). Uneven development in Accra has produced a fragmented urban landscape characterized by concentration of the poor in some locations, persistent pockets of affluence, and a patchwork of formal and informal growth along with increasing congestion, worsening environmental conditions, and growing inequalities in access to essential services like transportation, health facilities, educational institutions, and public utilities (Fuseini & Kemp, 2015; Korah et al., 2019).

## Methods

To understand how household-level resources and neighbourhoods shape development outcomes in a dense urban environment, we examine differences in SES groups outcomes when they are in the “in-group,” or surrounded by like households, and when they are in the “out-group” (i.e., the “stranger” group) when they are surrounded by different SES households. Testing how these “stranger” groups fare shows how or whether living in a poor area reinforces the disadvantages of the poor, as well as whether the resources of the affluent can ensure they meet basic standards of living. In this section we first discuss how we develop household SES categories and neighbourhood SES classes and then briefly introduce the outcomes of interest we track in our data. Finally, we discuss the statistical modelling strategy used to separately assess household compared to neighbourhood associations with the outcomes of interest.

### Defining household- and neighbourhood-based consumption categories

#### Defining household-based consumption categories

Since the census does not include income information, we use small area estimation methods (SAE) to “borrow strength” from Ghana Living Standards Survey (GLSS6) and predict consumption for census enumerated households. In collaboration with the Ghana Statistical Service (GSS), we applied this method to the 100% 2010 Population and Housing Census microdata and developed a dataset with poverty and inequality measures spatially identifiable at the enumeration area level (see the Methods Appendix). Since the GLSS does not sample the unhoused nor school-, hospital-, or prison-based populations, these are not included in this analysis. These household and area consumption metrics have been used in analyses elsewhere (Alli et al., 2023; Bixby et al., 2022; Clark et al., 2022; Tetteh et al., 2022). For this paper, we use these consumption estimates to determine the number of households that fall into three SES categories within each EA in Accra: households in the bottom 20^th^ percentile (poor), 21^st^-79^th^ percentiles (middle class), or the upper 20^th^ percentile (affluent). These categories represent the relative number of resources that households use to meet their needs (and wants).

We have three reasons for using relative as opposed to absolute measures of poverty and affluence. First, although the national poverty line is set based on those unable to meet their food and non-food needs, there is a general tendency to underestimate urban poverty since the high cost of living within the city is not factored into to the determination of poverty levels (Owusu & Yankson, 2007; Songsore, 2008; Owusu & Wrigley-Asante, 2020). Second, in a context that has been improving absolute levels of poverty, we chose a relative assessment of poverty to understand the ability of those at the bottom to meet their basic needs relative to their neighbours. A focus on the lower 20^*th*^ percentile group (“poor”) indicates a population that must make choices and trade-offs to prioritize certain needs over others. Put another way, households in this “poor” group consume at least 40% less than the median level of consumption in Accra. Third, our estimates of consumption are modelled. Predicted consumption estimates are most confidently interpreted in a relative sense, removing the need for calibration of the distribution of predicted values to an externally valid dataset of absolute consumption, an exercise that is fraught with challenges. We tested model sensitivity using alternative SES thresholds for the top/bottom 10^th^ and 30^th^ percentiles, which did not change the qualitative outcomes of our findings.

#### Defining neighbourhood-based consumption categories

We define neighbourhood SES for each EA using the Index of Concentration at the Extremes (ICE), a metric that measures how segregated an area is as the degree to which an area’s population is concentrated into extremes of poor or affluent (Massey, 2001; Krieger et al., 2017). The ICE metric is defined as *ICE_i_* = (*H_i_* – *L_i_*)/*T_i_* where *H_i_* is the number of people living in high-income (top 20^th^ percentile) households in area *i*, *L_i_* is the number of people in low-income (bottom 20^th^ percentile) households, and *T_i_* is the total number of individuals in the area. ICE values range from -1 to 1, with negative values indicating concentrated poverty, and higher values concentrations of affluence. Values around zero suggest areas could have a more even mix of households or a more homogenous middle class. ICE quantifies extreme concentrations of household types with one metric, identifying areas that are most polarized. Here we define neighbourhood-based SES using ICE tertiles: EAs in the bottom third are considered poorer neighbourhoods, while EAs at the top third of the ICE distribution are classified as affluent neighbourhoods. We tested model sensitivity to alternative ICE categories defined by the top/bottom ICE deciles or top/bottom ICE quintiles as well.

### Defining Dependent Variables: Improved Outcomes

We evaluate 16 “improved” outcome metrics as dependent variables that represent 5 different domains: housing quality, energy, sanitation, water, and information and communication technology (ICT) use. Definitions for “improved” versus “unimproved” come directly from UN’s Sustainable Development Goal guidelines (WHO-UNICEF, 2017; UNESCO, 2021; ITU, 2023) which have been used in past literature (e.g., MacTavish et al. 2023) although are not without criticism (cf. Herrera 2019, Weststrate et al., 2019) (see Appendix Table A.1 for additional details). These metrics indicate whether a household has ownership or access to certain materials or services, but cannot speak to reliability, availability, quality, or cost. Housing quality and the other service outcomes are related to defense mechanisms that protect residents from life- and health-threatening pollutants, pathogens, and other environmental and social risks (Songsore & McGranahan, 1993). ICT outcomes are related to health as increased access to information can have a positive effect on the usage of health services (Abekah-Nkrumah et al., 2014).

In the models presented below, the dependent variable is the percentage of households with improved outcomes within an SES category. In the housing quality domain, we include dwelling type, and wall, roof type, and flooring material. For energy use we examine the use of improved lighting sources and cooking fuels. Sanitation outcomes include use of improved toilets, liquid waste, and solid waste disposal. Drinking water outcomes are disaggregated into percent of households with piped, vended, and other improved sources to show variations in water use. ICT outcomes provide information on individuals with mobile phones or access to the internet, and households that have access to home computers and fixed landlines. Access to improved outcomes for some of these variables are contingent on household purchasing power, while others, such as those related to neighbourhood amenities are more related to decisions made by private services and public authorities.

### Modelling Strategy

Our goal is to model how differences in rates of improved household outcomes are explained by household SES versus neighbourhood SES. Notably, our approach aims to separate out whether better living conditions are associated with household or neighbourhood SES. It is beyond the scope of this paper to develop models that attempt to explain associations with improved outcomes through various covariates, rather we focus on independent associations with household and neighbourhood SES, and the interaction between them.

Outcomes for each SES group are summarized at the EA-level as proportions. We follow Papke and Wooldridge’s (1996) quasi-likelihood approach to estimate a generalized linear model using a logit-link function which respects the (0, 1) range of the dependent variable. The expected value of the improved outcome *E*(*y*|*x*) is estimated using a logistic function, and then parameters are estimated using Bernoulli quasi-likelihood methods. The advantage of this approach is that it does not assume any underlying structure to obtain *y*, only requiring that the conditional mean is specified correctly to ensure the predictions are bound between 0 and 1.

Our core model is specified as follows:

(1) $$Y_{ij}^{q}=\beta_{0}+\beta_{1}H_{ij}+\beta_{2}N_{j}+\beta_{3}H_{ij}N_{j}+\varepsilon_{ij}$$

where the *Y_ij_* is the proportion of households with improved outcome *q* for household SES category *i* in enumeration area *j*, *H_i_* is the household SES category and *N_j_* denotes neighbourhood SES category as indicated by its ICE value. *β*_1_ estimates the independent effect of households’ SES on the proportion with an improved outcome; *β*_2_ accounts for the same at the neighbourhood level. *β*_3_ estimates the effect of the interaction of household and neighbourhood SES (*H_ij_N_j_*) to jointly account for the type of household living in a specific neighbourhood type. We estimate this model in STATA 16 (StataCorp, 2019) using the glm function with the binomial family, logit link, and a robust variance estimator.

### Interpreting Model Results

The model presented in eq. 1 estimates household versus neighbourhood effects on improved outcomes independently and jointly via the interaction term. We use two prediction methods to estimate independent effects of household versus neighbourhood SES while also accounting for their joint interactions: average marginal effects (AMEs) and average adjusted predictions (AAPs).

AMEs help us assess whether household SES or neighbourhood affluence is more strongly associated with improved outcomes. AMEs around 0 suggest little difference relative to the middle-income group. AAPs are a regression-adjusted response variable, which allows us to interpret model results for different scenarios. To obtain AAPs, we use the fitted model to predict the margin (average improved outcome rate) for each group of interest (SES x Location) by fixing values of the covariates (Williams, 2012). AAPs allow us to estimate expected values for an outcome for each household SES category for each neighbourhood ICE tertile. We spatially map the outcomes of AAPs for poor and affluent household in poor and affluent neighbourhoods.

## Results

We first present a description of the spatial distribution of poverty and affluence across Accra and briefly describe spatial segregation in the city. We then present results by topical domain (housing, energy, sanitation, water, and ICT) using AMEs and AAPs to answer our research questions.

### Descriptive results

**Figure 2:**
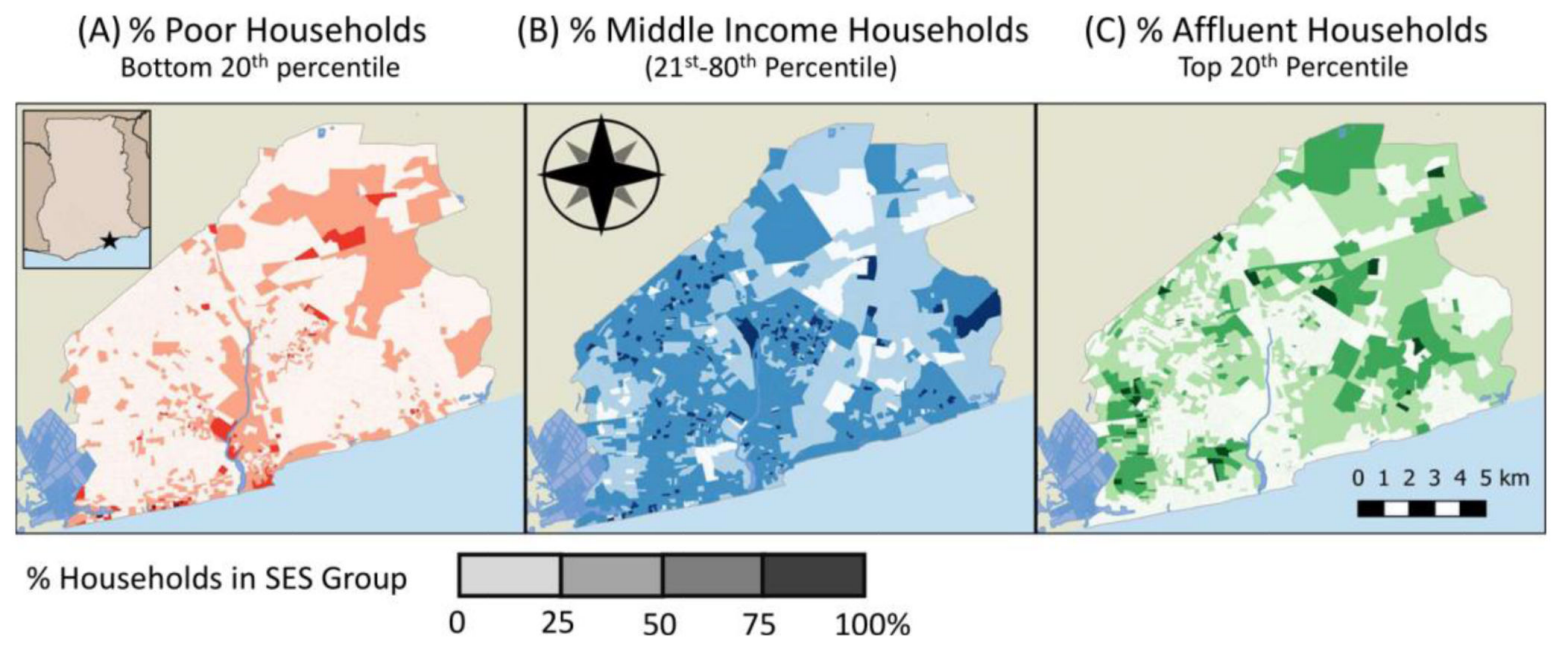
The percent of households in an EA that are (a) poor, (b) middle income, and (c) affluent across Accra.

Our data contain 501,851 households and 1,776,839 people living in 11 sub-metropolitan districts and 2,136 EAs in Accra. Across Accra, while poor and affluent households exist in most neighborhoods, rates of poverty and affluence vary significantly (Figure 2). There are pockets of poverty in the urban core, in and surrounding the traditional CBD, extending up through industrial areas along the Odaw River. There are high levels of concentrated poverty along the coastline, particularly near the Densu Delta in the west. There are high levels of poverty near the University of Ghana, and in areas near the airport. While many EAs with high rates of poverty are in historically vulnerable neighborhoods, such as Nima, Agbogbloshie, and Chokor, many are outside of places traditionally considered deprived. Clusters of affluence are often directly adjacent to poor areas in the core. Prominent affluent EAs can be seen in planned western neighbourhoods such as Dansoman, Mamprobi & Kaneshie. East of the Odaw, there are high rates of affluence in low density neighborhoods in a corridor that spans from the government Ministries to old colonial-era planned neighborhoods to the newly developed CBD near the airport. There are also high rates of affluence near the university. The middle SES group is spread throughout Accra, but is most prominent in unplanned communities near Nima and New Town as well as the Burma Camp military installation.

**Figure 3:**
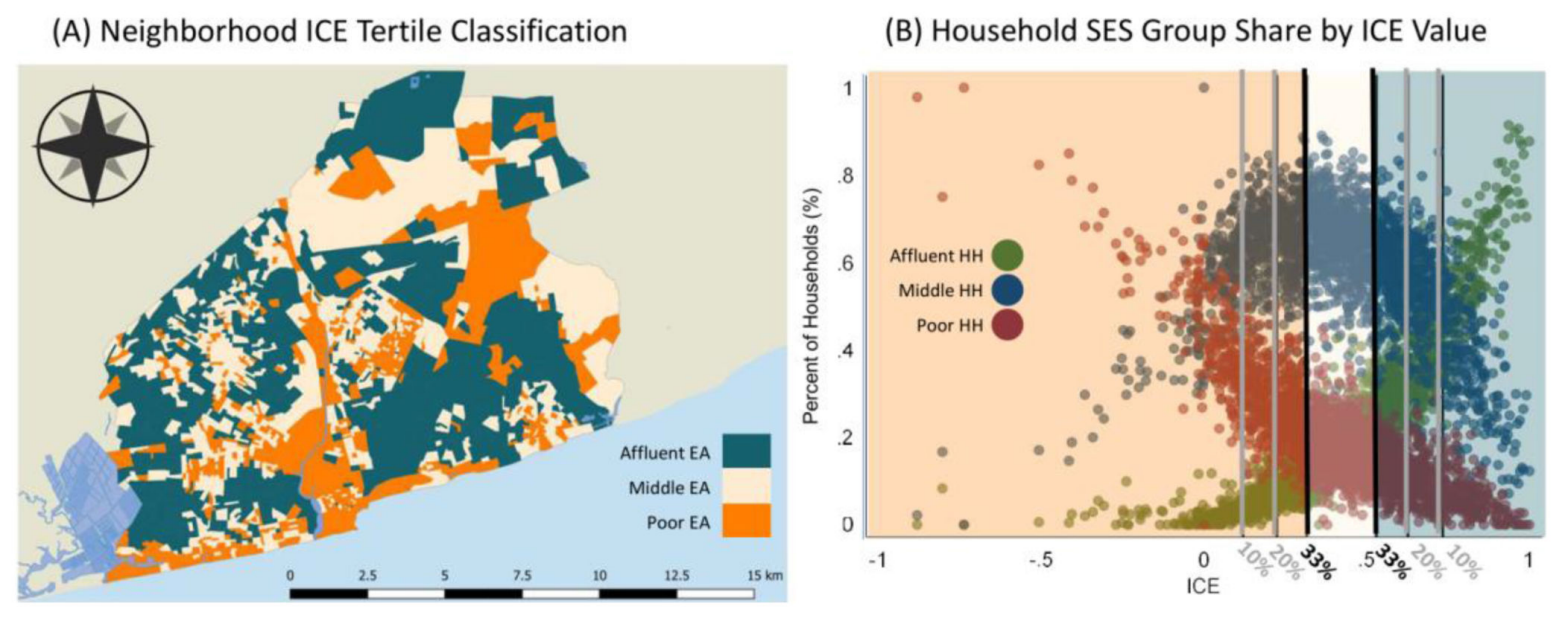
Segregation across Accra EAs. Panel A shows the ICE classification of each EA, and Panel B shows the EA percentage share of each SES group by ICE value. Black lines indicate tertile threshold boundaries, with grey marking alternative thresholds tested for robustness.

While some neighborhoods show highly concentrated poverty or affluence, neighborhoods often contain a mix of classes (Figure 3). Figure 3 shows the extent to which these neighborhoods are polarized, with orange areas showing areas that are dominated by poor households and dark green areas shows those that are highly affluent. This map reflects the geography of the distributional maps, however, there is greater polarization in poor areas than affluent areas. The beige EAs show areas where there are a high proportion of middle-income households or relatively equal mixes of poor and affluent households. These include middle SES areas like Burma Camp and Tema and neighborhoods to the west of the Odaw, and mixed areas like those near the university and those on the border of affluent and poor clusters. Similar to other LMIC countries, poverty is concentrated in core areas, and affluence is concentrated in historically affluent neighbourhoods and recently developed areas (van Ham et al., 2021).

Table 1 provides a description of poor and affluent households living in poor versus affluent neighbourhoods (EAs). Several characteristics are notable. At the household level, while about one-third of all households are female-headed, the highest rates are in poor households in poor areas (41%). Poor households are also more likely to live rent-free (22% and 37% in poor and affluent areas, respectively) (typically in informal settlements or supported by family members), while affluent households rent their homes at higher rates (51% and 52% in affluent and poor areas, respectively). In Accra, engagement in agriculture is rare except for affluent households within poor EAs. Characteristics of individuals in these areas show that poor households have a greater number of children under the age of 14 (34-35%) while affluent households have a greater proportion of working-age adults. There are educational differences as well, such as poor households have greater proportions that are uneducated (12-23%) or have just a basic education (36-38%), and affluent households are much more likely to have a post-secondary education (30-39%). However, we also see that while poor households in poor EAs have lower rates of secondary education than other groups (37%), poor households in affluent EAs keep up with affluent households (48%).

Regardless of SES, poor neighborhoods have far greater concentrations of Muslim households (20-27%), while affluent neighborhoods are predominantly Christian (85-90%) supporting the idea that other characteristics also influence residential self-selection.

### Household SES versus neighbourhood average SES

**Figure 4:**
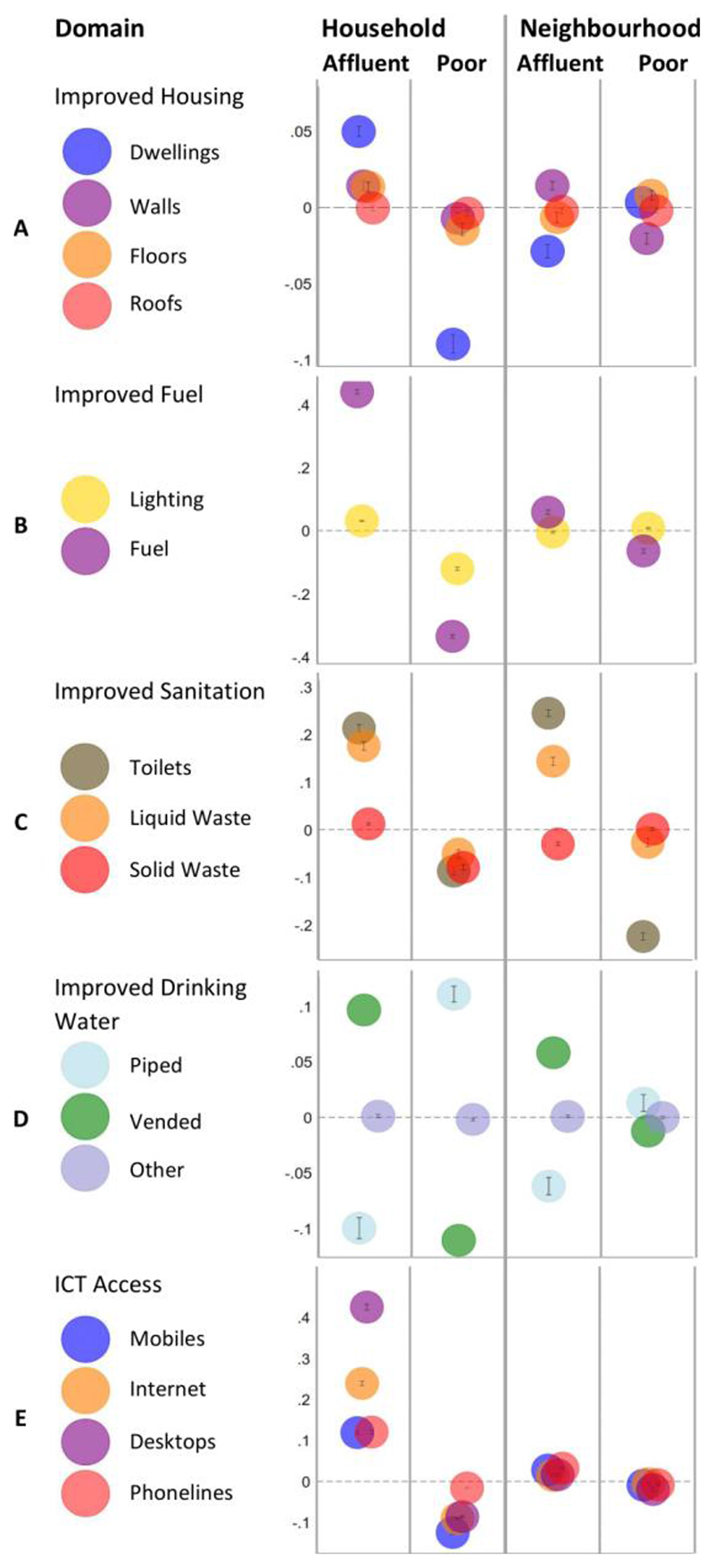
Average Marginal Effects (AMEs) for five household outcome themes. Each column shows the independent effect of an improved outcome as associated with household- or neighborhood-level SES.

Figure 4 presents average marginal effects (AMEs) from equation 1 for improved household outcomes (for tabular results see Table A.2). We use AMEs to assess whether household SES or neighbourhood SES is more strongly associated with improved outcomes, while accounting for their joint interaction. From these we can see whether household SES or location-based concentrations of SES are more associated with improved living conditions. A point on each figure represents the AME for household SES (*H_i_*) or neighbourhood SES (*N_j_*) as they relate to improved well-being outcomes grouped into five categories: housing (A), fuel use (B), sanitation (C), water (D), and ICT use (E). Since each outcome is calculated separately, axes are scaled to reflect the range of AMEs in that domain.

Row A presents outcomes related to housing: improved dwellings, floors, walls, and roofs. All rates showing in Row A are small relative to effects in other panels but, most noticeably, affluent (+) and poor (-) households are strongly associated with rates of improved dwellings (relative to middle income households). Most location-based marginal effects are smaller. Row B shows AMEs for improved energy sources, in which household SES is closely associated with the use of improved cooking fuels while location effects have a more limited relationship. Row C presents AMEs for sanitation outcomes. Here we see that household SES status is associated with improved toilets and liquid waste disposal, but in contrast to many other domains, location effects are of a very similar magnitude. Row D shows detailed results for piped, vended, and other drinking water sources. Household SES effects are strongly negatively related for piped water sources, showing affluent households are much more likely to use vended (bottled or sachet-packet) water sources. A similar though slightly smaller effect is seen for EAs. Finally, Row E presents AMEs related to ICT use, where we see strong household effects but virtually no relationship with one’s neighbourhood. These relationships persisted when testing model sensitivity with different thresholds (10% and 20% vs 33%; Fig 2B). The magnitude of class and neighbourhood effects increased with stricter definitions, and while levels decreased with more lenient cut-offs.

In summary, there are two main takeaways from the AME results. First, household SES is closely associated with consumption of improved housing materials, energy use, solid and liquid waste disposal services, drinking water, and ICT use – all things that are universally available and are aspects of living conditions over which households have a great degree of choice. Second, for other key domains, notably water and sanitation (improved toilets, liquid waste disposal services, and wall materials), location is as or more important than SES since these rely to some extent on publicly supplied infrastructure or markets that might be concentrated in affluent areas (e.g., vended water suppliers).

### How strangers fare: interactions between household and neighbourhood SES

**Figure 5:**
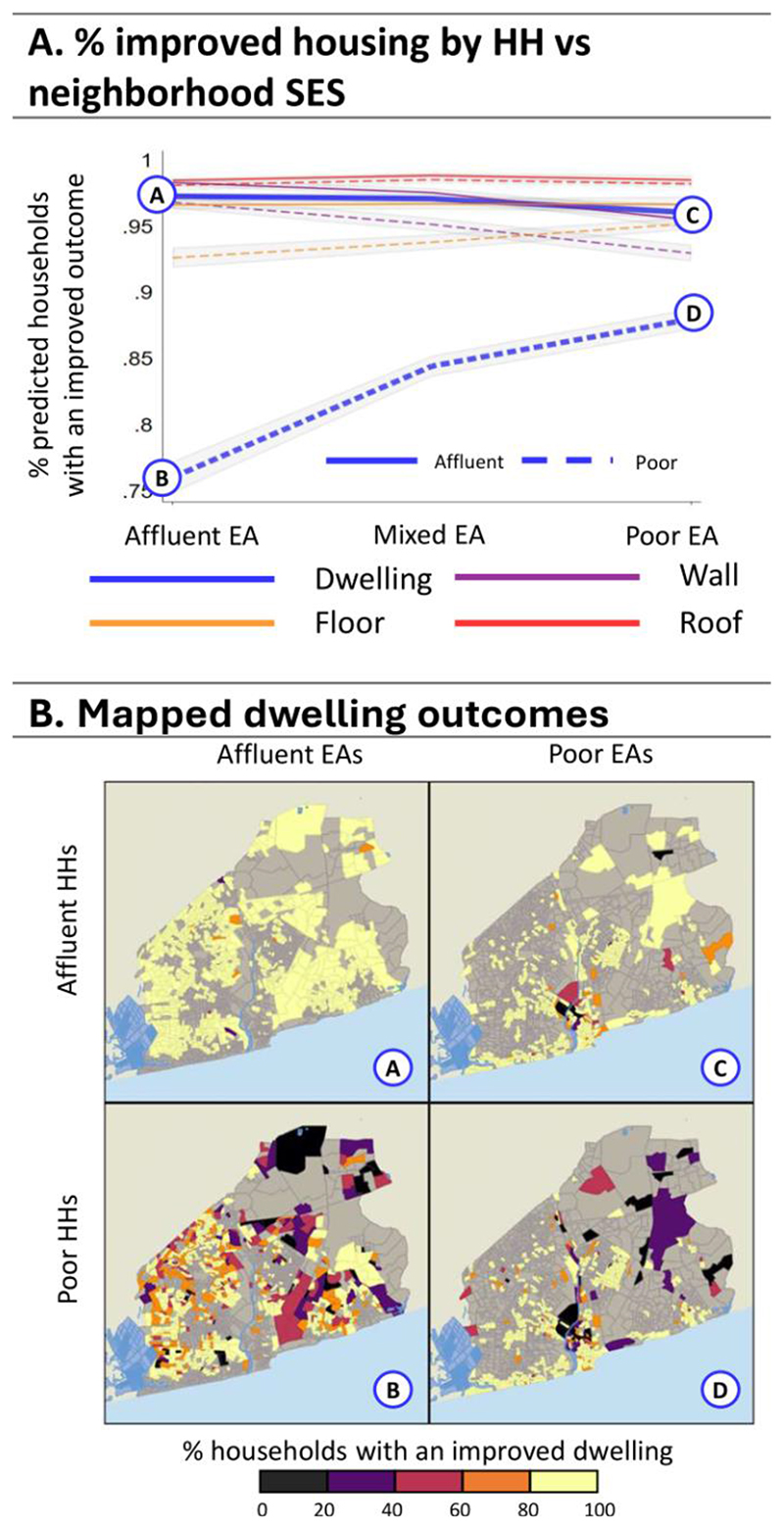
Average Adjusted Predictions for Improved Housing. Panel A: The predicted % of households with ‘improved’ measures for four housing-related variables for the affluent (solid line) vs poor (dashed lines) households in affluent, mixed, and poor EAs. Panel B: Maps A, B, C, and D show the spatial distribution associated with improved dwellings (points A, B, C, and D, respectively) from the line graph in Panel A.

**Figure 6:**
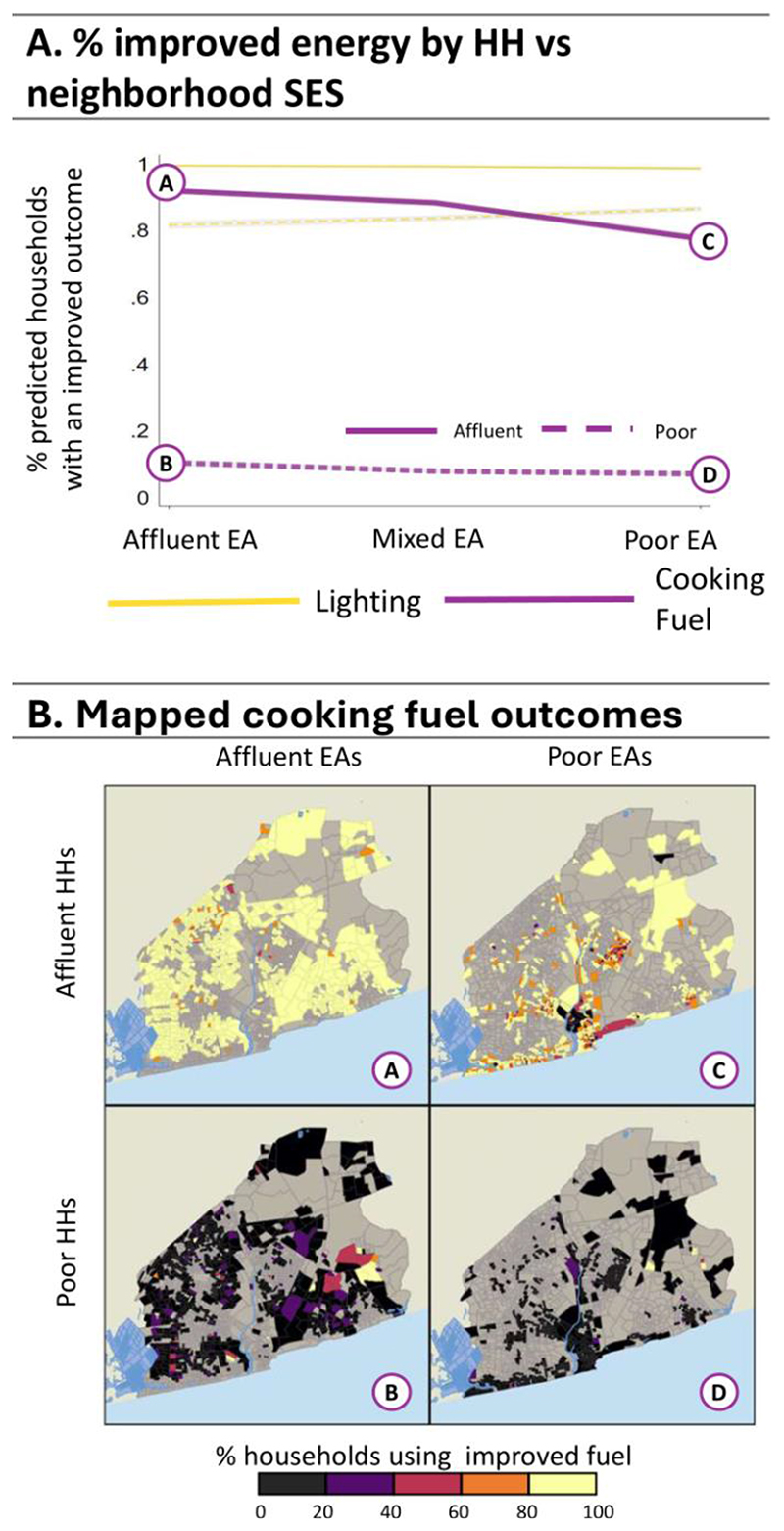
Average Adjusted Predictions for Improved Energy. Panel A: The predicted % of households with ‘improved’ measures for lighting and cooking fuel for the affluent (solid line) vs poor (dashed lines) households in affluent, mixed, and poor EAs. Panel B: Maps A, B, C, and D show the spatial distribution associated with improved cooking fuel (points A, B, C, and D, respectively) from the line graph in Panel A.

**Figure 7:**
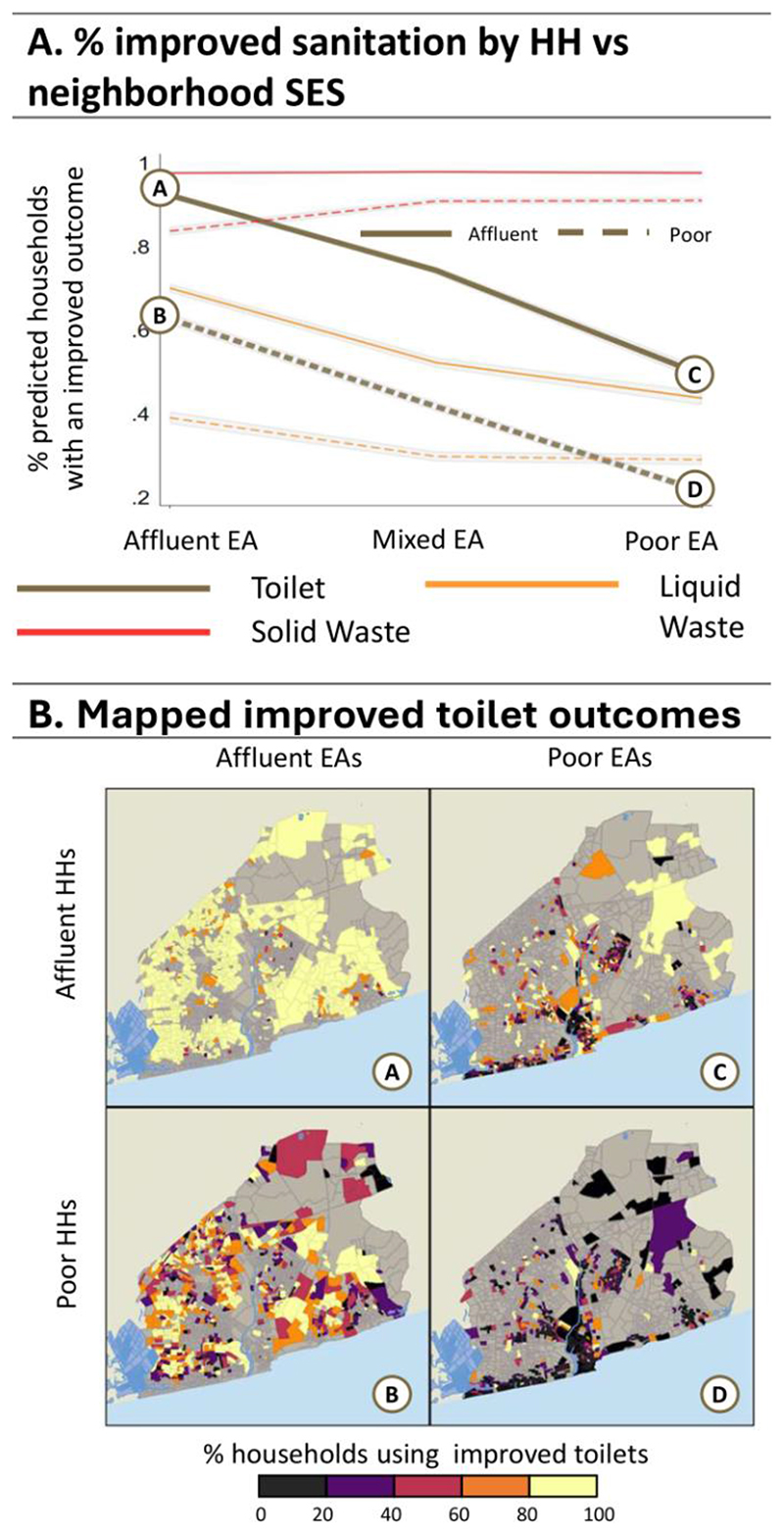
Average Adjusted Predictions for Improved Sanitation. Panel A: The predicted % of households with ‘improved’ sanitation measures for the affluent (solid line) vs poor (dashed lines) households in affluent, mixed, and poor EAs. Panel B: Maps A, B, C, and D show the spatial distribution associated with improved toilets (points A, B, C, and D, respectively) from the line graph in Panel A.

**Figure 8:**
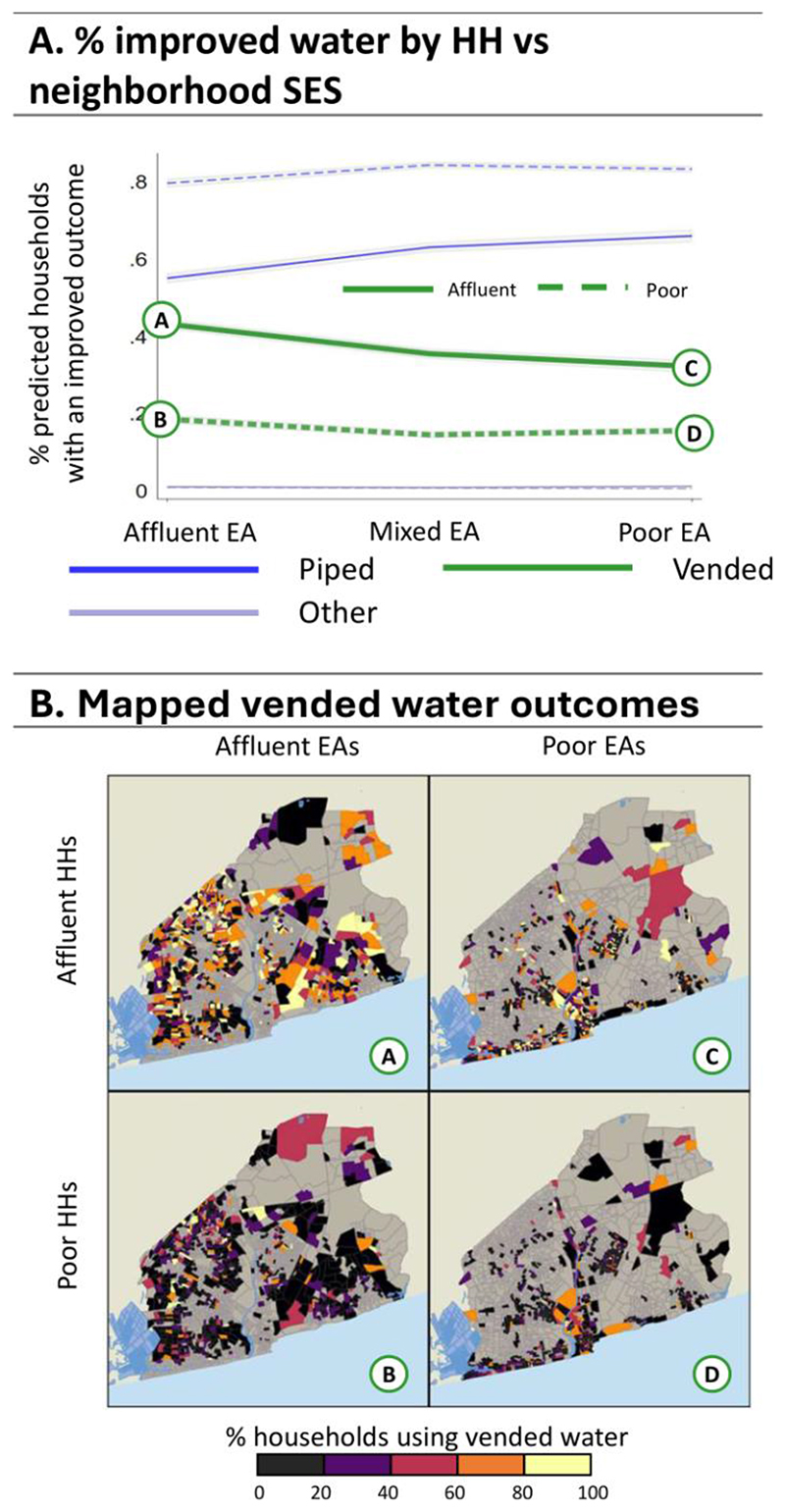
Average Adjusted Predictions for Improved Water. Panel A: The predicted % of households with water sources for the affluent (solid line) vs poor (dashed lines) households in affluent, mixed, and poor EAs. Panel B: Maps A, B, C, and D show the spatial distribution associated with vended water (points A, B, C, and D, respectively) from the line graph in Panel A.

We now turn to average adjusted predictions (AAPs) to see how households living in specific neighbourhood types might fare, presented in Figures 4-8 (see Appendix Table A.2 for regression estimates). In each figure, panel A shows a line graph of the average predicted percent of affluent (solid line) or poorer (dashed line) households with improved outcomes across the three neighbourhood EA types. Panel B presents a four-quadrant map of how outcomes for these groups differ spatially for one highlighted outcome (noted by the corresponding bolded line and letter markers in the graph). Each map quadrant shows the local rate of improved outcomes for a subset of the population. The top left row shows the spatial distribution of outcomes for affluent households in affluent EAs – corresponding to the situation at point (A) in the line graph above. Similarly, the top right row shows the distribution for affluent households in poor EAs, as in point (C), above. The bottom row shows outcomes for poor households in affluent (B) and poor neighbourhoods (D).

Figure 5 presents AAPs for improved housing-related outcomes, with the maps highlighting improved dwellings specifically. AAPs show that across all household and neighbourhood types, more than 90% of households have improved outcomes for walls, floors, and roofs. For dwelling types, however, the poor have much *worse* outcomes in affluent EAs (76.01%) relative to in poor EAs (87.95%), suggesting that having affluent neighbours is not related to improved dwelling conditions for the poor. Spatially, we see most affluent households (Fig 5.A and Fig 5.C) have very high rates of improved dwellings, save for those who live in industrial areas in the urban core. Poor households (Fig 5.B and Fig 5.D) have lower rates of improved dwellings. The poor have very low rates in affluent areas around the AMA, particularly in planned EAs. In poor areas, low rates of improved dwellings are highly concentrated in the urban core, and near newly developed areas, but most areas do very well. Overall, these results suggest, perhaps counterintuitively, the poor have on average better housing conditions in poorer neighbourhoods.

Figure 6 presents AAPs for improved energy use. Disparities are noticeably large in affluent versus poor households for use of improved cooking fuel where, on average, 85.8% of the affluent and only 8.4% of the poor use improved fuels. Poor households also have worse outcomes in poor areas compared to affluent ones, but the neighbourhood effects here are small. Outcomes for lighting are a much more better as most affluent (99%) and poor households (83%) have improved lighting. Again, however, the poor have better lighting outcomes in poor neighbourhoods (86%) than affluent areas (81%) Spatially, the maps highlight that disparities in cooking fuel are strongly associated with household and not neighbourhood type, although affluent households in poorer core EAs have poorer outcomes than elsewhere.

Figure 7 presents AAPs for improved sanitation outcomes on three metrics: solid waste disposal, liquid waste disposal, and improved toilet access, with the maps highlighting access to improved toilets. While use of improved solid waste disposal methods are common (95% of affluent and around 90% of poor households), only 40% and 53% of households have improved liquid waste disposal and toilets in the AMA on average. These are strongly patterned by both household and neighbourhood SES. Improved toilet rates for the affluent are higher in affluent EAs (92%) than in poor EAs (50%). Similarly, the poor have higher improved toilet rates in affluent EAs (62.5%) as compared to poor EAs (21%). This is our only result where the poor living in an affluent area fare better than affluent households living in poor areas. The maps show that there is a distinct spatial component where there are low rates of improved toilet access in the urban core poor areas regardless of household SES. In affluent neighborhoods, where the affluent have very high access, there is greater variation in access to improved toilets for the poor, with the lowest rates appear in EAs that border poor areas.

Figure 8 shows AAPs for sources of drinking water with the quadrant map focusing on vended sources. Piped drinking water use among affluent households is highest in poor EAs (65.9%) and lowest in affluent EAs (54.9%). Vended water fills in these gaps, with the highest rates in affluent EAs (43%) and the lowest in poor EAs (32%). Poor households rely on piped water to a much greater degree, ranging from 79.5% in affluent EAs to 83% in poor EAs. Spatially, in affluent neighborhoods, there is greater use of vended water with affluent households than poor households indicating there is service but affluent households choose to purchase water. However, this is highly variable in outlying neighborhoods. In poor EAs, particularly those near the coast, there are very low rates of households using vended water, however vended water rates increase in affluent households further out from the core.

**Figure 9:**
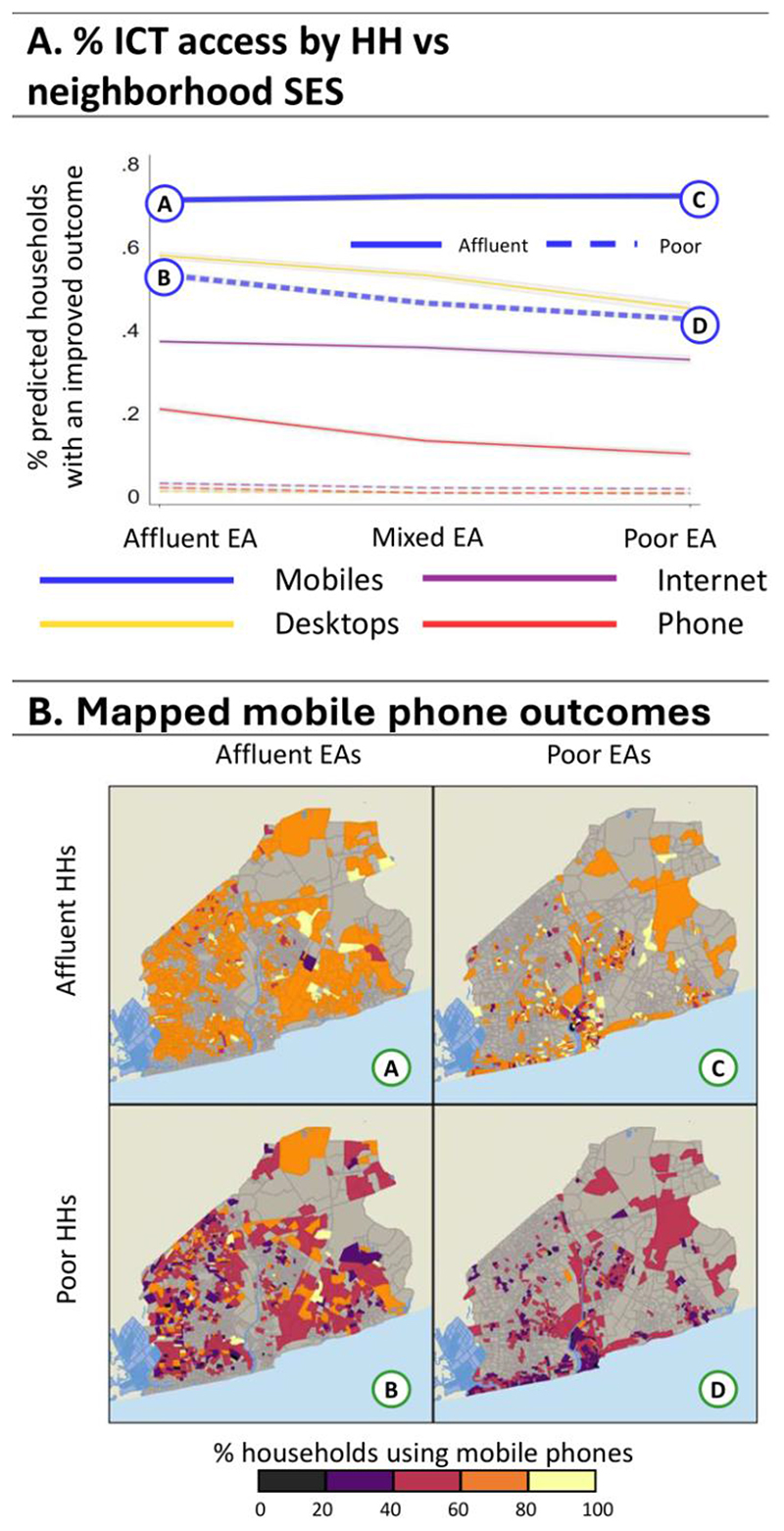
Average Adjusted Predictions for ICT Use. Panel A: The predicted % of households with ICT use for the affluent (solid line) vs poor (dashed lines) households in affluent, mixed, and poor EAs. Panel B: Maps A, B, C, and D show the spatial distribution associated with mobile phone use (points A, B, C, and D, respectively) from the line graph in Panel A.

Figure 9 shows AAPs for information, communication and technology (ICT) outcomes regarding mobile phone, internet use, desktop ownership, and presence of a fixed land lines. In general, affluent groups have much better ICT outcomes than all SES groups, as also reflected in Figure 4. Around 70% of affluent households have a mobile phone, regardless of neighbourhood, while about 57%, 49% and 45% of poor households have a mobile in affluent, middle, and poor neighbourhoods, respectively. For other ICTs, affluent households in affluent EAs have the highest rates, and declines in poor EAs. Among other ICTs, the poor have all have very low rates of use. Spatially, there is a lot of homogeneity in affluent EAs, and the poor have the high rates of access near the airport and university. In poor EAs, affluent households have high rates of mobile use except in Agbogbloshie, while usage drops for poor households in the urban core and the wester coastline.

The key takeaway from AAP results is that, even for the areas where SES matters more (i.e., as demonstrated in Figure 5), location and household interactions can have major effects on outcomes. Concentrated areas of affluence or poverty can improve or worsen outcomes for ‘stranger’ households, though it seems the direction of these effects are somewhat unpredictable. In some instances, living in an affluent EA is associated with greater rates of improved outcomes for the poor, but there are also cases where living in an affluent EA seems to worsen outcomes for poor households. Conversely, there are situations where living in a poor EA can worsen circumstances for affluent households. We discuss these results and their implications for policy design in the next section.

## Discussion

### Household versus neighbourhood effects

Household consumption levels influence household budgets and therefore choices they make, but neighbourhoods also shape the services and markets to which households have access. Table 2 summarises the household and neighbourhood effects, with summary implications for development policy, taking into account different ways household and neighbourhood status interact for measures of well-being. The resulting outcomes fall into four general patterns. Case A covers most of the outcomes measured – household SES has a positive association with improved wellbeing metrics, and generally neighbourhoods pull stranger’s outcomes closer to their mean (poor households do better in affluent areas, affluent households do worse in poor areas). For floors and piped water – case B – the poor in affluent neighbourhoods have worse outcomes and the affluent in poor neighbourhoods have better outcomes. In case C, strangers always have worse outcomes in a stranger neighbourhood. Case D represent outcomes that show no relationship with household or neighbourhood SES.

Understanding how household SES and neighbourhoods interact for improved outcomes is important for directing limited resources to ameliorate deprivation in different areas of concern. When household SES dominates, policy can target households based on household SES or socio-demographic traits. For example, Ghana’s Livelihood Empowerment Against Poverty (LEAP) cash transfer program targets the most vulnerable of households (Wodon, 2012; Cuesta et al., 2019). Alternatively, when neighbourhoods relate more strongly to deprivation, policy makers can direct resources and programming in a more location-based way.

Neighbourhood effects capture aspects of public services, markets, and environmental quality to which nearby households have access, which are often unevenly distributed. Affluent, high-status areas can attract investment for further development and services infrastructure, while older, poorer neighbourhoods more often repel capital flows (Gottlieb, 1997; Krätke, 2014). Concentrations of poverty can reproduce and reinforce disadvantages, though policy might implement place-based programs that can target such marginalized areas (Partridge & Rickman, 2008; Barca et al., 2012). To reduce disparities in domains that are particularly affected by location (e.g., sanitation), improving infrastructure and capacities in the most deprived places may make for effective public investment (Barca et al. 2012). Other than public infrastructure investment, location-oriented policies might include regulating private operators to prevent or subsidize unmanageable price increases for basic needs in poor communities or to ensure operators of critical services provide adequate and complete coverage over all neighbourhoods (Appiah-Effah et.al., 2019; Oteng-Ababio et al., 2013).

### Application to drinking water

A complementary household- and neighbourhood-based strategy would be useful in contending with Accra’s water challenges. The two dominant sources of drinking water are through the public piped network and through vended (purchased) water outlets (Moulds et al., 2022; Tetteh et al., 2022). Affluent, planned communities such as Airport Residential Areas, Ridges, and Cantonments often have better access to the public water supply network than poorer communities, which influences access and pricing (Mahama et al., 2014; Tetteh et al., 2022). While most households have access to improved drinking water, the Ghana Water Company has not been able to meet growing demand and thus must ration water delivery at times (Stoler, et al., 2013; Tetteh et al., 2022). Further, rationing can be spatially inconsistent – some neighbourhoods receive water every day while in other areas delivery is sporadic or non-existent (Dapaah & Harris, 2017). Inconsistency in piped water delivery carries an additional risk since negative water pressures risk seepage and cross contamination with raw sewage (Stoler et al., 2013). Thus, piped water has become something of an inferior good and vended water is perceived as the safer, healthier choice (Stoler et al., 2014). According to Moulds et al., 2022, over half of urban households in the region who use sachet water as their primary source also have a piped supply connection.

Our results indicate poor households are highly reliant on piped water, while affluent households are more likely to use vended water. Previous work has found that when households turn to vended sources outside of the piped system due to rationing and health concerns, affluent households are more likely to use bottled water, and poorer households are likely to purchase sachet water (Moulds et al., 2022; Tetteh et al., 2022). In areas particularly plagued by unmaintained infrastructure and rationing, poor households must choose to use vended water despite the high markup (Moulds et al., 2022; Stoler et al. 2014; Tetteh et al., 2022). However, there are many households for which that is not a feasible strategy as it would put too much pressure on limited budgets.

To address these challenges programs could help make vended water affordable to vulnerable households in areas where infrastructure is limited, strengthen, and maintain the water supply system to ensure safety and prevent loss of public potable water supply, or regulate private sector water provision to ensure equitable and safe service across the city. In areas where there are high rates of vended water use amongst the affluent, but not the poor, indicates there may be issues with the quality of piped water (allowing those who can afford to choose the better option to do so). Other areas, such as nearby the university, have low rates of vended water use amongst the affluent and point to trust in the quality of water, and high vended water rates amongst the poor. This indicates that access to connecting to piped-water infrastructure may be a major barrier for poorer households. The former case may require investment in infrastructure to improve safety and quality of a more affordable water source. The latter example may need cash assistance or expansion of the existing system to reach households and provide water at a more affordable price. Appropriate targeting of such policies would be best informed by understanding household and neighbourhood interactions.

### Implications for mixed development

How well does Massey’s (2001) assertion that ‘living in a poor area reinforces disadvantages of the poor, and living in affluent neighbourhoods strengthens the advantages of the affluent’ hold up in a LMIC context? In many cases our results generally support the assertion. Poor in poor areas have worse outcomes in use of improved cooking fuel, toilets, vended water use, and all ICT metrics. Similarly, affluent household living in affluent areas have better outcomes in dwelling types, cooking fuel, all sanitation outcomes, and vended water, and ICT outcomes (though mobile ownership is constant).

However, Massey’s assertion is far from a universal truth. Table 2 suggests that moving a poor household from a poor area to a middle income or affluent neighbourhood only signals an improvement in 9 out of 16 cases, only slightly better than half of our wellbeing outcomes. In contrast, for a number of housing characteristics (dwelling type, floors, roofs) as well as solid waste disposal and lighting, poor households in poor areas have *better* outcomes than they do in affluent areas. This may indicate that “pockets” of poverty within affluent areas may be particularly precarious or informal, or costs to improved housing and services in affluent areas may be even more prohibitive than they are in poorer settlements. In these areas concentrated affluence worsens outcomes for ‘stranger’ households since the poor may not have the same community resources, social network, or access to services that often underpin welfare and community health. On the other hand, in most cases being affluent in a poor area is associated with poorer outcomes than they would have in a affluent neighbourhood: moving to a poor area decreases affluent well-being in 11 out of 16 indicators. Overall promotion of mixed-income neighbourhoods is likely good for a number of outcomes, as shown in Table 2.A. Mixed development may be further justified for a number of dynamic and intergenerational reasons (e.g., Chetty et al., 2014, 2016, 2022), however, in some cases it can exacerbate inequality (Table 2.B) or result in worse outcomes for stranger households (Table 2.C). Targeted policies to aid vulnerable communities, regardless of where they live, are still needed. For example, poor households in affluent areas where infrastructure is available would benefit from policies focused on providing assistance in accessing these services.

This study has some data limitations that also help put our results in context. To model the aggregate effects of household versus neighborhood characteristics, we abstract from contextual factors that could be important determinants of the outcomes we investigate and operate *through* the household or neighborhood. We also do not take an explicit spatial regression approach so we do not control for clustering of neighborhood types. Instead, we account for space using random effects. Finally, the patterns we assess are the product of individual household residential choices. As such, our results are indicative of empirical patterns but are not causal estimates. Still, these patterns and results help us see what outcomes are possible, and thus likely, when promoting mixed-use development.

## Conclusion

Good quality housing is crucial to ensuring household welfare and health; poor housing and environmental conditions put residents at risk of health problems such as infectious diseases, stress, and depression (Yakubu et al., 2014). Poor quality, overcrowded, and badly located housing not only influences physical health, but mental well-being which affects workforce participation and educational attainment (Yakubu et al., 2014). Overall housing conditions are affected by type of dwelling, construction materials, household facilities and the coverage of neighbourhood services (Gibson et al., 2011). ICT access has significant implications for potential educational attainment and access to formal banking services. Examining the disparities in housing quality and service provision can inform policy efforts in Accra. Identifying how household SES and neighbourhood effects jointly influence improved outcomes can help decision makers choose how to direct resources via area-based or population-based programs. While 2010 data reflects the Accra as it was ten years ago, this analysis serves as a baseline for future analysis of the just completed 2021 census.

We find household SES is more closely related to higher rates of improved housing materials, energy use, solid waste disposal services, drinking water, and ICT use. In these cases, policy makers might be well served to focus on household-level barriers to consumption of these services. A household’s location is as or more important than household SES in the case of walls, improved toilet use, improved disposal of liquid waste, and vended water use. In these cases, policy makers may consider an area-based approach that target particularly deprived areas. Finally, our results suggest that location interacts with household status – often further advantaging affluent households in affluent areas or increase disadvantages experienced by poor households in poor areas and being a ‘stranger’ in one location brings you closer to that location’s mean. Still, we also find cases where being poor in an affluent neighbourhood is associated with larger disadvantages – namely for housing related outcomes – suggesting pockets of poverty may present particularly isolated and marginalized living conditions. Ultimately, both personal cost as well as infrastructural or neighbourhood-level barriers stymie efforts to address most environmental and health challenges. As such, it is important to identify where these issues are most critical between and among household SES groups to give policy makers a more accurate portrait of disparities.

## Supplementary Material

Appendix

## Figures and Tables

**Table 1:** Population characteristics

	Affluent EA (712)		Poor EA (712)	
	Affluent hh (57,631)	Poor hh (15,034)	Affluent hh (11,275)	Poor hh (54,581)
**Household characteristics**				
***Head of Household***				
Female-headed household	32%	36%	30%	**41%**
***Housing Tenure***				
Owner occupied	34%	25%	34%	36%
Renting	**51%**	32%	**52%**	40%
Rent-free	15%	**37%**	14%	**22%**
Other	1%	5%	1%	3%
***Agrarian***				
Engaged in agriculture	8%	1%	**11%**	0%
Crops	68%	63%	63%	**80%**
Trees	9%	4%	**10%**	5%
Livestock	22%	33%	26%	**15%**
Fish	1%	1%	1%	1%
Non-agricultural household	92%	99%	89%	100%
**Individual characteristics**	229,785	45,397	42,517	199,256
***Age***				
0-14	23%	**34%**	22%	**35%**
15-64	**72%**	61%	**74%**	60%
64 plus	5%	5%	4%	5%
***Religion***				
Muslim	6%	9%	**20%**	**27%**
Christian	**90%**	**85%**	77%	65%
Other religions	2%	2%	1%	1%
No religion	1%	4%	2%	6%
***Nationality***				
Ghanaian	94%	98%	94%	97%
Foreign	6%	2%	6%	3%
***Education***				
Never schooled	3%	**12%**	5%	**23%**
Ever schooled	97%	88%	95%	77%
Basic education	17%	**36%**	18%	**38%**
Secondary	41%	48%	47%	37%
Post-secondary	**39%**	4%	**30%**	2%
***Employment Sector***				
Main employment	53%	52%	55%	52%
Primary	1%	1%	1%	2%
Secondary	9%	18%	10%	13%
Tertiary	42%	33%	43%	36%
Unemployed	47%	48%	45%	48%

*Note: Bold indicates values that are notably larger or smaller than other groups within that domain*

**Table 2:** How do stranger groups fare in strange neighbourhoods?

	Household SES effect	Stranger effect		Development implications
		Poor in affluent n’hood	Affluent in poor n’hood	
**A. Strangers’ outcomes are closer to group outcomes**. The poor in affluent neighbourhoods have better outcomes; the affluent in poor neighbourhoods have worse outcomes.				
*Toilets*				**Mixed development reduces inequality**
*Liquid Waste*				
*Walls*				
*Vended water*				
*Fuel*				
*Mobiles*				
*Phone lines*				
*Desktops*				
*Internet*				
**B. Marginalization exacerbated**. The poor in affluent neighbourhoods have worse outcomes; the affluent in poor neighbourhoods have better outcomes.				
*Floors*				**Mixed development exacerbates inequality**
*Piped Water*				
**C. Stranger disadvantage**. Strangers do worse (or no better) in stranger neighbourhoods.				
*Dwelling*				**Mixed development makes all worse off (only hh income matters)**
*Lighting*				
*Solid Waste*				
**D. No relation**. SES and neighbourhood have no association with improved outcomes.				
*Roofs*				**No impact**
*Other Water*				

*Notes: Green indicates a positive and statistically significant effect, red denotes a negative and statistically significant effect, grey denotes effects indistinguishable from zero (Fig 5-9). Darker shading indicates which effect has a larger AME (Fig 4).*
