# Supplementary material for: Strangers in a strange land: mapping household and neighbourhood associations with improved wellbeing outcomes in Accra, Ghana
Source: Cities. Author manuscript; Available in PMC 2023 Dec 1. (PMC7615188; doi:10.1016/j.cities.2023.104584)
Supplement: Appendix [file EMS189115-supplement-Appendix.pdf]

Appendix

SAE Methods Description

Our data are uniquely derived from the 100% 2010 Population and Housing Census (PHC) collected by the GSS. This procedure is documented in detail in Cavanaugh et al., 2022. The Ghanian census is spatially identifiable at the EA-level (on average ~10,000 m^2), however, the census does not contain income or consumption data that are typically used as an indicator of a household's socioeconomic status (SES). In the developing context, this is often due to expense of collecting data and difficulty obtaining answers. Even when surveys include this information, collected data is only available for larger geographical units or the sample size is not large enough to produce accurate estimates (Nyugen et al., 2017). Since we do not have information on income, we measure consumption. While income is preferred when examining economic standards of living, the benefit of using consumption is that it is an appropriate measure of "someone's actual standard of living regardless of how it is attained" (Johnson et al., 2005). The GLSS6 provides information on many categories of household expenditures, and expenditures including rent is used as our measure for household consumption.

To estimate consumption for households with the 100% census, we first use another dataset – the Ghana Living Standards Survey (GLSS6), which is measured in detail – to develop a statistical relationship between consumption and common predictors that capture demographics, education, employment, and housing conditions (see below for additional detail on these). Notably, these predictors are also available in exactly the same format in the census.

Following the small area estimation (SAE) literature (Elbers et al., 2003; Molina & Rao 2010), we first fit a linear mixed model with area-level random intercepts to

GLSS6 survey data. Our dependent variable is consumption divided by the square root of household size to account for household-level economies of scale (Buhmann et al. 1988), SAE methods that “borrow strength” from the detailed information in the GLSS6 and apply it to the more spatially detailed and representative census data Using the parameter estimates from the GLSS6 consumption model, we then predict consumption for the full census data. We simulate this model 100 times, drawing from variance around parameter estimates and assign the average of the simulated values as the consumption.

Appendix References

Buhmann, B., Rainwater, L., Schmaus, G., & Smeeding, T. M. (1988). Equivalence scales, well-being, inequality, and poverty: sensitivity estimates across ten countries using the Luxembourg Income Study (LIS) database. *Review of income and wealth*, *34*(2), 115-142.

XXXX [anonymized by the authors] (2022). Locating poverty and inequality: an application of small area estimation methods using survey and census data from Ghana. Manuscript in preparation.

Elbers, C., Lanjouw, J. O., & Lanjouw, P. (2003). Micro-level estimation of poverty and inequality. Econometrica, 71(1), 355-364.

Johnson, D. S., Smeeding, T. M., & Torrey, B. B. (2005). Economic inequality through the prisms of income and consumption. Monthly Lab. Rev., 128, 11.

Molina, I., & Rao, J. N. K. (2010). Small area estimation of poverty indicators. *Canadian Journal of Statistics,* 38 (3): 369-385.

Nguyen, M. C., Corral, P., Azevedo, J. P., & Zhao, Q. (2017). Small area estimation: An extended ELL approach. World Bank.

Appendix Table A.1. Improved/Unimproved Household Characteristic Classification

	Improved	Unimproved
Housing		
Dwelling type	Compound house (rooms)	Huts/Buildings (different compound)
	Flat/Apartment	Huts/Buildings (same compound)
	Semi-detached house	Improvised home (kiosk/container, etc.)
	Separate house	Living quarters attached to office/shop
		Other
		Tent
		Uncompleted building
Wall material	Burnt bricks	Bamboo
	Cement blocks/Concrete	Mud brick/Earth
	Landcrete	Other
	Metal sheet/Slate/Asbestos	Palm leaf/Thatch (grass)/Raffia
	Stone	Wood
Floor material	Burnt brick	Earth/Mud
	Cement/Concrete	Other
	Ceramic/Porcelain/Granite/Marble tiles	Wood
	Stone	
	Terrazzo/Terrazzo tiles	
	Vinyl tiles	
Roof material	Cement/Concrete	Bamboo
	Metal sheet	Mud/Mud bricks/Earth
	Roofing tile	Other
	Slate/Asbestos	Thatch/Palm leaf or Raffia
		Wood
Energy		
Lighting	Electricity (mains)	Candle
	Electricity (private generator)	Crop residue
	Solar energy	Firewood
		Flashlight/Torch
		Gas lamp
		Kerosene lamp
		Other
Cooking Fuel	Electricity	Animal waste
	Gas	Charcoal
	Kerosene	Crop residue
		None, no cooking
		Other
		Saw dust
		Wood

Appendix Table A.1 continued. Improved/Unimproved Household Characteristic Classification

	Improved	Unimproved
Sanitation		
Toilet	KVIP	Bucket/Pan
	Pit latrine	No facilities (bush/beach/field)
	W.C.	Other
		Public toilet (WC, KVIP, Pit, Pan, etc.)
Liquid waste disposal	Through drainage into a pit (soak away)	Other
	Through drainage system into a gutter	Thrown into gutter
	Through the sewerage system	Thrown onto compound
		Thrown onto the street/outside
Solid waste disposal	Collected	Buried by household
	Public dump (container)	Burned by household
	Public dump (open space)	Dumped indiscriminately
		Other
Drinking Water Source		
Pipe-borne	Pipe-borne inside dwelling	Dugout/Pond/Lake/Dam/Canal
	Pipe-borne outside dwelling	Other
Vendor	Bottled water	Rainwater
	Sachet water	River/Stream
Other	Bore-hole/Pump/Tube well	Tanker supply/Vendor provided
	Protected spring	Unprotected spring
	Protected well	Unprotected well
	Public tap/Standpipe	
ICT		
Mobile Phone	Owns mobile phone	Does not own mobile phone
Internet Access	Accesses the internet	Does not access the internet
Desktops	Household has desktop or laptop	Household has no desktop or laptop
Fixed phone line	Household has fixed phone line	Household has no fixed phone line

Appendix Table A.2. GLM Regression Results

obs. = 40,798
EA= 2136

**Improved Living Conditions**	SES Aff.	SES Poor	ICE Aff.	ICE Poor	SES#ICE Aff.#Aff.	SES#ICE Aff.#Poor	SES#ICE Poor#Aff.	SES#ICE Poor#Poor	Intercept	AIC	BIC	Log pseudolikelihood
Housing												
Dwelling	0.973*	-0.826*	-0.261*	-0.038	0.327*	-0.273*	-0.274*	0.338*	2.514*	0.487	-421432	-9919.800
Wall	0.542*	-0.156*	0.488*	-0.407*	-0.120	-0.214*	-0.056	0.017	3.120*	0.301	-424732	-6120.713
Floor	0.371	-0.278	-0.138	0.208	0.121	-0.213	-0.047	0.065	2.986	0.33	-424120	-6721.978
Roof	0.091	-0.156	-0.106	-0.1	-0.171	-0.153	-0.135	-0.084	4.314	0.134	-429654	-2730.524
Energy												
Lighting	1.577	-1.473	-0.034	0.16	0.344	-0.645	-0.111	0.064	3.099	0.340	-425231	-6933.437
Fuel	2.350*	-2.123*	0.310*	-0.291*	0.097*	-0.487*	0.004	0.17*	-0.340*	0.886	-413765	-18053.303
Sanitation												
Solid Waste	0.255*	-1.235*	-0.654*	0.076	0.532*	-0.182	-0.001	-0.057	3.517*	0.324	-424154	-6591.175
Liquid Waste	0.787*	-0.160*	0.627*	-0.069*	0.132*	-0.273*	-0.215*	0.03	-0.7*	1.101	-404741	-22443.625
Toilet	1.104	-0.290	1.227	-0.975	0.181	-0.111	-0.375	0.011	-0.047	0.959	-412273	-19545.729
Drinking Water												
Piped	-0.452*	0.694*	-0.2901*	0.08*	-0.042	0.048	-0.028	-0.157*	0.983*	0.991	-407377	-20214.71
Vendor	0.459*	-0.718*	0.286*	-0.072*	0.038	-0.074	0.010	0.158*	-1.059*	0.969	-407669	-19746.87
Other	-0.054	-0.279*	0.034	-0.079	0.157	0.37	0.209	-0.14	-4.716*	0.087	-430738	-1760.847
ICT												
Mobiles	0.577*	-0.512*	0.108*	-0.011	-0.152*	0.017	0.168*	-0.147*	0.369*	0.919	-427772	-18730.41
Internet	1.523*	-1.77*	0.155*	0.009	-0.093*	-0.139*	0.278*	-0.15*	-2.11*	0.550	-426206	-11216.269
Desktops	2.381*	-2.569*	0.111*	-0.093*	0.079*	-0.227*	0.287	0.179	-2.256	0.537	-422470	-10938.709
Phone line	1.989*	-0.991*	0.820*	-0.183*	-0.274*	-0.119	0.168	-0.032	-3.863*	0.278	-426849	-5655.583

** denotes statistical significance at the .05 level*

Estimated in Stata using specification suggested by Baum (2008): glm Y i.SES##EA_SES , link(logit) family(binomial) vce(robust)
